# Homeoprotein Neuroprotection of Embryonic Neuronal Cells

**DOI:** 10.1523/ENEURO.0061-19.2019

**Published:** 2019-09-26

**Authors:** Stephanie E. Vargas Abonce, Mélanie Leboeuf, Alain Prochiantz, Kenneth L. Moya

**Affiliations:** 1 Centre for Interdisciplinary Research in Biology (CIRB), Collège de France, CNRS UMR 7241/INSERM U1050, PSL Research University, Labex Memolife Paris Science et Lettres, 75005 Paris, France; 2 BrainEver, 75012 Paris, France

**Keywords:** DNA damage, homeoprotein, neuroprotection, transcription factor

## Abstract

Most homeoprotein transcription factors have a highly conserved internalization domain used in intercellular transfer. Internalization of homeoproteins ENGRAILED1 or ENGRAILED2 promotes the survival of adult dopaminergic cells, whereas that of OTX2 protects adult retinal ganglion cells. Here we characterize the *in vitro* neuroprotective activity of several homeoproteins in response to H_2_O_2_. Protection is observed with ENGRAILED1, ENGRAILED2, OTX2, GBX2, and LHX9 on midbrain and striatal embryonic neurons, whereas cell-permeable c-MYC shows no protective effects. Therefore, five homeoproteins belonging to three different classes (ANTENNAPEDIA, PAIRED, and LIM) share the ability to protect embryonic neurons from midbrain and striatum. Because midbrain and striatal neurons do not express the same repertoire of the four proteins, a lack of neuronal specificity together with a general protective activity can be proposed. Interestingly, hEN1 and GBX2 provided protection to primary midbrain astrocytes but not to non-neural cells, including mouse embryo fibroblasts, macrophages or HeLa cells. For the four proteins, protection against cell death correlated with a reduction in the number of H_2_O_2_-induced DNA break foci in midbrain and striatal neurons. In conclusion, within the limit of the number of cell types and homeoproteins tested, homeoprotein protection against oxidative stress-induced DNA breaks and death is specific to neurons and astrocytes but shows no homeoprotein or neuronal type specificity.

## Significance Statement

Homeoproteins are DNA binding proteins regulating gene expression throughout life. Many of them transfer between cells and are thus internalized by live cells. This has allowed their use as therapeutic proteins in animal models of Parkinson’s disease and glaucoma. Part of their therapeutic activity is through a protection against neuronal death. Here we show that internalized homeoproteins from three different classes protect embryonic ventral midbrain and striatal neurons from oxidative stress, at the level of both DNA damage and survival. The interest of this finding is that it lends weight to the possibility that many homeoproteins play a role in neuroprotection through shared mechanisms involving, in particular, DNA protection against stress-induced breaks.

## Introduction

Homeoprotein (HP) transcription factors, discovered on the basis of their developmental functions, remain expressed in the adult where they exert not fully understood physiologic activities (Di Nardo et al., 2018). Several HPs transfer between cells thanks to highly conserved secretion and internalization domains present in their DNA binding site or homeodomain (HD). HP internalization has allowed for the use of OTX2, ENGRAILED1 (EN1), and ENGRAILED2 (EN2; collectively ENGRAILED), as therapeutic proteins in animal models of Parkinson’s disease (ENGRAILED) and glaucoma (OTX2; Sonnier et al., 2007; Alvarez-Fischer et al., 2011; Torero-Ibad et al., 2011; Thomasson et al., 2019).

The two proteins EN1 and EN2 are expressed in adult midbrain dopaminergic (mDA) neurons (Di Nardo et al., 2007). These neurons degenerate progressively in parkinsonian patients, in classical Parkinson’s disease animal models and in the *En1*-heterozygous mouse. In all models tested, ENGRAILED injected or infused is internalized by mDA neurons and prevents their death, even following a strong and acute oxidative stress provoked by a 6-hydroxydopamine hydrobromide (6-OHDA) injection at the level of the substantia nigra pars compacta (SNpc; Rekaik et al., 2015). The mechanisms involved in this protection have started to be analyzed. ENGRAILED internalization stimulates the translation of complex I mitochondrial proteins, restores the chromatin epigenetic marks disrupted by the stress, and allows for DNA repair, as quantified by the number of γH2AX foci (Alvarez-Fischer et al., 2011; Rekaik et al., 2015). In addition, ENGRAILED represses the expression of LINE-1 mobile elements caused by oxidative stress *in vitro* and *in vivo* (Blaudin de Thé et al., 2018). Because of the epigenetic nature of the protection mechanisms, a single injection of ENGRAILED has long-lasting effects, including in nonhuman primates (Thomasson et al., 2019), opening the way for a therapeutic use of this HP.

In view of developing ENGRAILED as a therapeutic protein, human EN1 (hEN1) was produced and purified; and an assay was adapted to test hEN1 for neuroprotection against oxidative stress and, in particular, to evaluate protein activity, specificity, and stability. Because OTX2 has a similar survival effect on mDA neurons and retinal ganglion cells (RGCs; Torero-Ibad et al., 2011; Rekaik et al., 2015), it could be that protection against oxidative stress is a shared property of several HPs with little HP and/or neuronal specificity. To test this hypothesis, the protective effect of EN1, EN2, OTX2, GBX2, and LHX9 was evaluated on midbrain and striatal neurons in culture as well as testing hEN1 on a neuronal cell line. We show that the five proteins, but not cell-permeable c-MYC, protect embryonic midbrain and striatal neurons against oxidative stress-induced cell death and DNA damage caused by H_2_O_2_ but are ineffective on mouse embryo fibroblasts (MEFs), peritoneal macrophages, and HeLa cells. hEN1 was also protective against H_2_O_2_ oxidative stress in primary midbrain astrocytes and against 6-OHDA in the dopaminergic LUHMES (Lund human mesencephalic) cells. The protective activity in the different cell types and against the two stressors involves reducing DNA damage.

## Materials and Methods

### Animal treatment

All animals were treated in accordance with the applicable guides, directives, and authorizations for the care and use of laboratory animals.

### Cell cultures

For neuronal primary cultures, pregnant Swiss mice (Janvier) were killed by cervical dislocation 14.5 d postconception (dpc), and the embryos were extracted and placed in PBS-glucose 0.6%. Striatal or midbrain structures were dissected in 2 ml of PBS-glucose, and cells were mechanically dissociated and plated at a density of 25,000 cells/well in poly-l-ornithine (15 μg/ml for glass coverslips and 1.5 μg/ml for plastic wells) and 2.5μg/ml laminin-coated 96-well plates (Sigma-Aldrich) for LDH assay and 24-well plates with glass coverslips for immunocytochemistry. Cells were cultured in Life Technologies Neurobasal medium (Thermo Fisher Scientific) supplemented with glutamine (500 μm; Sigma-Aldrich), glutamic acid (3.3 mg/L; Sigma-Aldrich), aspartic acid (3.7 mg/L; Sigma-Aldrich), Gibco Antibiotic-Antimycotic (anti-anti; Thermo Fisher Scientific), and Gibco B27 (Thermo Fisher Scientific; NB^+^) for 24 h at 37°C in a humidified incubator with 5% CO_2_ atmosphere. All experiments were performed at 6 d *in vitro* (DIV).

Primary astrocytes were prepared from postnatal day 1 Swiss mice (Janvier) killed by decapitation. The midbrain was dissected, and the cells were mechanically dissociated. Cells were grown in 25 cm^2^ tissue culture flasks at 37°C with 5% CO_2_ atmosphere in DMEM/F-12, Life Technologies GlutaMAX supplement (Thermo Fisher Scientific), supplemented with 10% (v/v) Gibco fetal bovine serum (FBS; Thermo Fisher Scientific), high glucose, 5 mm Gibco HEPES (Thermo Fisher Scientific), and Gibco anti-anti (Thermo Fisher Scientific). Cells at 80% confluence were detached with 0.05% Gibco Trypsin-EDTA (Thermo Fisher Scientific), replated as above until reaching 80% confluence, trypsinized, seeded on 96-well plates at a density of 15,000 cells/well in the same culture medium, and cultured for 24 h before stopping proliferation with cytosine arabinoside (1 μm Ara C). This led to nearly pure astrocyte cultures, based on glial fibrillary acidic protein expression.

Primary MEFs were isolated from the skin of 11 dpc Swiss mouse embryos (Janvier) according to Jozefczuk et al. (2012). Cells were grown on 75 cm^2^ tissue culture flask at 37°C in a humidified incubator with 5% CO_2_ atmosphere. Cells at 80% confluence were detached using 0.05% Gibco Trypsin-EDTA (Thermo Fisher Scientific), plated at a density of 12,500 cells/well in 96-well tissue culture plastic plates and cultured for 24 h in DMEM, high glucose, and Gibco GlutaMAX (Thermo Fisher Scientific) supplemented with 10% (v/v) Gibco FBS (Thermo Fisher Scientific) before the addition of 10 μm Ara C.

HeLa cells (RRID:CVCL_0030) were maintained in DMEM, 1 × g/L d-glucose l-glutamine, Gibco Pyruvate (Thermo Fisher Scientific) supplemented with 10% (v/v) Gibco FBS (Thermo Fisher Scientific). Cells were grown on 75 cm^2^ tissue culture flasks at 37°C in a humidified incubator with 5% CO_2_ atmosphere. Cells at 80% confluence were detached using 0.05% Gibco Trypsin-EDTA (Thermo Fisher Scientific) and plated at a density of 12,500 cells/well in 96-well tissue culture plastic plates. Cells were cultured for 24 h before stopping proliferation with 10 μm Ara C.

Macrophages were isolated from the mouse peritoneal cavity of 8-week-old Swiss female mice (Janvier). Mice were killed by cervical dislocation and peritoneal washes were performed using HBSS. After massaging the peritoneum, the fluid containing resident macrophages was collected, seeded, and plated at a density of 100,000 cells/well in 96-well tissue culture plastic plates in Gibco DMEM + GlutaMAX (Thermo Fisher Scientific) and 2% Gibco FBS (Thermo Fisher Scientific) at 37°C in a humidified incubator with 5% CO_2_ atmosphere.

LUHMES cells (catalog #CRL-2927, ATCC; RRID:CVCL_B056) were thawed rapidly at 37°C, transferred to a 15 ml Falcon tube with 3 ml of AdvDMEM, and centrifuged for 7 min at 190 × *g*. Supernatant was discarded, and 1 ml of DMEM was added to the pellet. After gentle resuspension, the cells were placed in AdvDMEM+FGF (40 ng/ml) and cultured for 3 d at 37°C before trypsinization (0.025% Trypsin 0.1 g/L and EDTA in PBS) for 5 min at 37°C, followed by the addition of 4 ml of AdvDMEM medium and centrifugation for 7 min at 190 × *g*. The cells were dissociated with 1 ml of Advanced (Adv) DMEM+FGF and plated at a density of 30,000 cells/well on 96-well plates previously coated with laminin (1 μg/ml) and poly-l-ornithine (50 μg/ml) for the LDH assay, or coated with laminin (1 μg/ml) and poly-l-ornithine (500 μg/ml) glass coverslips for immunocytochemistry. Cells were cultured in AdvDMEM+FGF for 1 or 3 d at 37°C in a humidified incubator with 5% CO_2_ atmosphere for the LDH assay or γ-H2AX foci analysis, respectively. Proteins ware added at the times indicated in the text in the presence 1 μm Ara C.

### qRT-PCR

Total RNA was extracted using the RNeasy Mini kit (Qiagen) and reverse transcribed using the QuantiTect Reverse Transcription kit (Qiagen). The qRT-PCR was made using SYBR-Green (Roche) and Light Cycler 480 (Roche). Data were analyzed using the “2-ddCt” method, and values were normalized to *Gapdh* (glyceraldehyde 3-phosphate dehydrogenase).

### Protein production

Chicken ENGRAILED2 (chEN2) and mutant chicken ENGRAILED2 (SR-EN2), mouse EN1 (mEN1), hEN1, and mouse OTX2 (mOTX2) were prepared as described previously (Joliot et al., 1998; Torero-Ibad et al., 2011). Cell-permeable recombinant human c-MYC was purchased from Abcam (catalog #ab169901), and human GBX2 (hGBX2) and human LHX9 (hLHX9) were purchased from Proteogenix. Endotoxins were removed by phase separation according to Aida and Pabst (1990). Unless stated otherwise, proteins were stored at −20°C.

### Protein treatment and oxidative stress

Cells were incubated with different concentrations of HPs diluted in culture media. For neutralization, HPs were preincubated with a 10-fold molar excess of antibody for 1 h at 37°C. For LDH and trypan blue assay, oxidative stress was induced by incubation for 2 h at 37°C in 50 mm H_2_O_2_ (Sigma-Aldrich) or 6-OHDA (300, 200, or 100 μm) diluted in culture media. For DNA break analysis, H_2_O_2_ (100 μm) or 6-OHDA (10 or 50 μm) were added for 1 h. For dye-exclusion survival analysis, the media were replaced with 0.16% trypan blue for 5 min at room temperature (RT) then replaced with PBS, and the number of cells excluding trypan blue or not were counted blind in five fields of view at 20×, five wells per condition. The LDH assay was conducted using the CytoTox 96 Non-Radioactive Cytotoxicity Assay (Promega) according to manufacturer instructions (Moravec, 1994).

### Immunocytochemistry

Coverslips were washed three times in PBS, fixed in 4% paraformaldehyde for 30 min at RT, washed in PBS three times, permeabilized with PBS/0.5% Triton (Sigma-Aldrich) for 45 min at RT and placed in 100 mm Glycine for 30 min at RT. After a 1 h incubation at RT in PBS/10% natural goat serum (NGS; Thermo Fisher Scientific)/1% Triton, primary antibodies were added overnight at 4°C. The next day coverslips were washed three times in PBS, incubated with secondary antibodies for 2 h at RT, washed three times in PBS, and mounted in DAPI Fluoromount-G (Southern Biotech). The mouse monoclonal anti-γH2AX antibody (IgG1) is from clone JBW301 (Millipore; RRID:AB_309864), the mouse monoclonal anti-β-tubulin III (Tuj 1 IgG2A) from clone SDL.3D10 (Sigma-Aldrich; RRID:AB_532291). The two primary antibodies were used at a 1:500 dilution, and Alexa Fluor-conjugated goat anti-mouse (RRID:AB_2535764) and goat anti-chicken (RRID:AB_2535781) antibodies (Thermo Fisher Scientific) at a 1:2000 dilution.

### Quantification of DNA damage

Images corresponding to a coverslip diameter were acquired with a Nikon i90 Microscope and exported to ImageJ. DNA damage was quantified by counting the number of γH2AX foci present in the nucleus of Tuj1^+^ cells. Counting was conducted blind to conditions on four coverslips per condition.

### Statistical analysis

Data are expressed as the mean ± SD if not otherwise indicated, and results were analyzed with GraphPad Prism version 6 (RRID:SCR_002798). For the trypan blue experiment, statistical significance was determined by one-way ANOVA and two-tailed *t* test using five wells per condition. For the LDH assay experiments, statistical significance was determined by one-way ANOVA and a *post hoc* Dunnett’s test for comparisons to H_2_O_2_ using eight replicates per condition (ns: nonsignificant, *p < 0.5, **p < 0.005, ***p ≤ 0.001 and ****p < 0.001 in all experiments). Statistical power (Table 1) for each significant difference was determined using the statistical power calculator (https://www.stat.ubc.ca/~rollin/stats/ssize/n2.html; accessed on 18–19 February 2019, 4 July and 24 July 2019, and 1 August 2019).

**Table 1.** Statistical power analysis

Data structure		Type of test	Power
Trypan blue	Control vs H_2_O_2_	*Post hoc t* test, two-tailed	1.00
	H_2_O_2_ vs 12.5 nm		0.92
Figure 1*A*	H_2_O_2_ vs 12.5 nm	Dunnett’s multiple comparisons	1.000
	H_2_O_2_ vs 2.5 nm		1.000
	H_2_O_2_ vs 25 pm		1.000
	H_2_O_2_ vs 12.5 pm		1.00
	H_2_O_2_ vs 2.5 pm		1.000
	H_2_O_2_ vs 1.25 nm		1.000
Figure 1*B*	H_2_O_2_ vs 12.5 nm	Dunnett’s multiple comparisons	1.000
	H_2_O_2_ vs 2.5 nm		1.00
Figure 1*C*	H_2_O_2_ vs 12.5 nm 1× frozen	Dunnett’s multiple comparisons	1.000
	H_2_O_2_ vs 2.5 nm 1× frozen		1.00
	H_2_O_2_ vs 12.5 nm 5× frozen		1.00
	H_2_O_2_ vs 2.5 nm 5× frozen		1.000
	H_2_O_2_ vs 12.5 nm 4°C		1.000
	H_2_O_2_ vs 2.5 nm 4°C		1.000
Figure 1*D*	H_2_O_2_ vs 12.5 nm chEn2	Dunnett’s multiple comparisons	1.000
	H_2_O_2_ vs 2.5 nm chEn2		1.000
	H_2_O_2_ vs 12.5 nm hEn1Q50A		1.000
	H_2_O_2_ vs 2.5 nm hEn1Q50A		1.000
	H_2_O_2_ vs 12.5 nm hEn2		1.000
	H_2_O_2_ vs 2.5 nm hEn2		1.000
Figure 2*A*	H_2_O_2_ vs 12.5 nm hEn1	Dunnett’s multiple comparisons	1.000
	H_2_O_2_ vs 2.5 nm hEn1		1.000
	H_2_O_2_ vs 12.5 nm mOtx2		1.000
	H_2_O_2_ vs 2.5 nm mOtx2		1.000
	H_2_O_2_ vs 12.5 nm hGbx2		1.000
	H_2_O_2_ vs 2.5 nm hGbx2		1.000
	H_2_O_2_ vs 12.5 nm hLhx9		1.000
Figure 2*B*	H_2_O_2_ vs 12.5 nm hEn1	Dunnett’s multiple comparisons	1.000
	H_2_O_2_ vs 2.5 nm hEn1		1.000
	H_2_O_2_ vs 12.5 nm mOtx2		1.000
	H_2_O_2_ vs 2.5 nm mOtx2		1.000
	H_2_O_2_ vs 12.5 nm hGbx2		1.000
	H_2_O_2_ vs 2.5 nm hGbx2		1.000
	H_2_O_2_ vs 12.5 nm hLhx9		1.000
	H_2_O_2_ vs 2.5 nm hLhx9		1.000
Figure 2*C*	H_2_O_2_ vs 12.5 nm hEn1	Dunnett’s multiple	1.000
	H_2_O_2_ vs 2.5 nm hEn1	comparisons	1.000
	H_2_O_2_ vs 12.5 nm hGbx2		1.000
	H_2_O_2_ vs 12.5 nm hLhx9		1.00
Figure 3*B*	H_2_O_2_ vs 2.5 nm mEn1	Dunnett’s multiple comparisons	1.00
	H_2_O_2_ vs 1.25 nm mEn1		1.000
	H_2_O_2_ vs 0.62 nm mEn1		1.00
	H_2_O_2_ vs 0.31 nm mEn1		1.00
	H_2_O_2_ vs 3.3 nm mOtx2		1.000
	H_2_O_2_ vs 1.65 nm mOtx2		1.00
	H_2_O_2_ vs 0.82 nm mOtx2		1.000
	H_2_O_2_ vs 0.41 nm mOtx2		1.00
	H_2_O_2_ vs 2.68 nm hGbx2		1.000
	H_2_O_2_ vs 1.34 nm hGbx2		1.000
	H_2_O_2_ vs 0.67 nm hGbx2		1.000
	H_2_O_2_ vs 0.33 nm hGbx2		1.000
	H_2_O_2_ vs 2.27 nm hLhx9		1.000
	H_2_O_2_ vs 1.14 nm hLhx9		1.000
	H_2_O_2_ vs 0.56 nm hLhx9		1.000
	H_2_O_2_ vs 0.28 nm hLhx9		1.000
Figure 3*C*	H_2_O_2_ vs stavudine	Dunnett’s multiple comparisons	1.000
	H_2_O_2_ vs hEn1		1.000
Figure 3*D*	H_2_O_2_ vs stavudine	Dunnett’s multiple comparisons	1.000
	H_2_O_2_ vs hEn1		1.000
Figure 4*A*	−hEN1 vs +hEN1 at 300 μm 6-OHDA	*Post hoc t* test, two-tailed	1.000
	−hEN1 vs +hEN1 at 200 μm 6-OHDA	*Post hoc t* test, two-tailed	1.000
Figure 4*B*	−hEN1 vs +hEN1 at 10 μm 6-OHDA	*Post hoc t* test, two-tailed	1.000
	−hEN1 vs +hEN1 at 50 μm 6-OHDA	*Post hoc t* test, two-tailed	1.000

## Results

### EN1 protects embryonic neurons against H_2_O_2_ oxidative stress

Five independent hEN1 (human), one mEN1 (mouse), and one chEN2 (chicken) preparations produced similar results. Protective activity was measured by the LDH cytotoxicity assay, except for one experiment in which trypan blue exclusion was used for comparison. In the trypan blue experiment, mean survival of embryonic day 14.5 (E14.5) ventral midbrain cells in the control condition was 89.3 ± 4.0%. Two hours after adding H_2_O_2,_ neuron survival was reduced to 36.5 ± 14.1% (*p* < 0.0001 compared with control). The survival of neurons pretreated with 12.5 nm hEN1 was significantly greater (66.4 ± 9.6%, *p* < 0.005) compared with H_2_O_2_-treated cells. The LDH assay gave qualitatively similar results and was thus used thereafter, making it easier to test different preparations and dose responses over a large range of HP concentrations.

**Figure 1.**
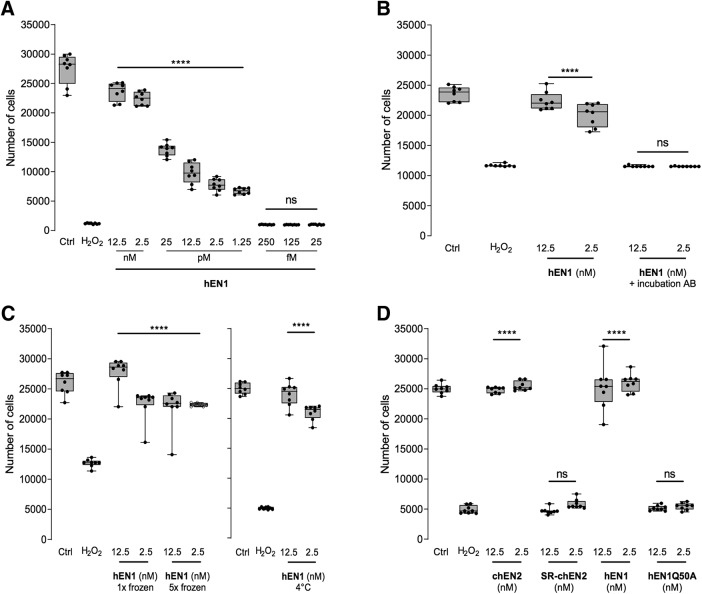
ENGRAILED protection of embryonic midbrain neurons. ***A***, hEN1 dose-dependent survival of embryonic neurons after H_2_O_2_ oxidative stress. ***B***, Preadsorption of hEN1 with an anti-ENGRAILED antibody abrogates hEN1 neuroprotection. ***C***, hEN1 subjected to repeated freeze–thaw cycles (left) or maintained at 4°C for 6 weeks (right) has significant neuroprotective activity against oxidative stress. ***D***, ENGRAILED internalization and high-affinity DNA binding are necessary for ENGRAILED neuroprotection. LDH assay was used for ***A–D***. ns, nonsignificant, ****p < 0.0001.

LDH is a cytosolic enzyme and this assay measures LDH released into the culture medium after the lysis of live cells. Figure 1*A* shows an hEN1 dose–response survival experiment for embryonic midbrain neurons at 6 DIV. Two hours after oxidative stress with 50 mm H_2_O_2_, >90% of the cells are dead. Pretreatment of the cells with 1.25 pm to 12.5 nm significantly increases their survival from ∼28% to 86% in an EN1 dose-dependent manner. It is of note that H_2_O_2_ effects were variable between experiments with oxidative stress-induced cell death varying between 50% and 90%. Based on this dose response, 12.5 and 2.5 nm HP concentrations allowing for total or near total protection were used in additional experiments.

Since hEN1 is a recombinant protein purified from bacterial extracts, its activity could be in part due to a contaminant. As shown in Figure 1*B*, protective activity was fully abolished by preincubation (1 h at 37°C) of the protein with an anti-EN1 polyclonal antibody (Alvarez-Fischer et al., 2011), establishing that the neuroprotective activity is entirely due to hEN1. To examine hEN1 stability, aliquots were frozen on dry ice and thawed one or five times. Midbrain neurons were treated with hEn1, and 24 h later with 50 mm H_2_O_2_. Human EN1 frozen and thawed once provided 100% protection and 84–70% protection if frozen and thawed five times (Fig. 1*C*, left). The protein maintained at 4°C for 6 weeks also retained full protective activity at 12.5 and 2.5 nm (Fig 1*C*, right).

Homeoprotein internalization is driven by the third helix of the homeodomain (Derossi et al., 1994) and within this sequence mutating tryptophan (W) in position 48 of the HD blocks internalization (Derossi et al., 1996). Accordingly, chEN2 internalization is abolished if the W and phenylalanine (F) residues at positions 48 and 49 of the HD are changed to serine and arginine (chEN2SR) residues, respectively (Joliot et al., 1998). Wild-type mEN1, hEN1, and chEN2 provided 100% protection against H_2_O_2_ oxidative stress while no protection was observed by chEN2SR (Fig. 1*D*), demonstrating that cell internalization is necessary for protection.

In addition to transcription, EN1 and EN2 also regulate protein translation (Brunet et al., 2005; Alvarez-Fischer et al., 2011; Stettler et al., 2012). Glutamate at position 50 of the homeodomain does not modify internalization but is necessary for high-affinity DNA binding and transcriptional activity (Le Roux et al., 1993). To determine whether ENGRAILED protective activity depended on transcription, hEN1 with a glutamine-to-alanine mutation at position 50 (hEN1Q50A) was produced. Figure 1*D* illustrates that, in contrast with wild-type hEN1, incubation with hEN1Q50A at the same concentrations provided no protection against H_2_O_2_ oxidative stress. This demonstrates that EN1 protection against oxidative stress requires both internalization and high-affinity DNA binding activity.

### Other HPs protect primary neurons against oxidative stress

**Figure 2.**
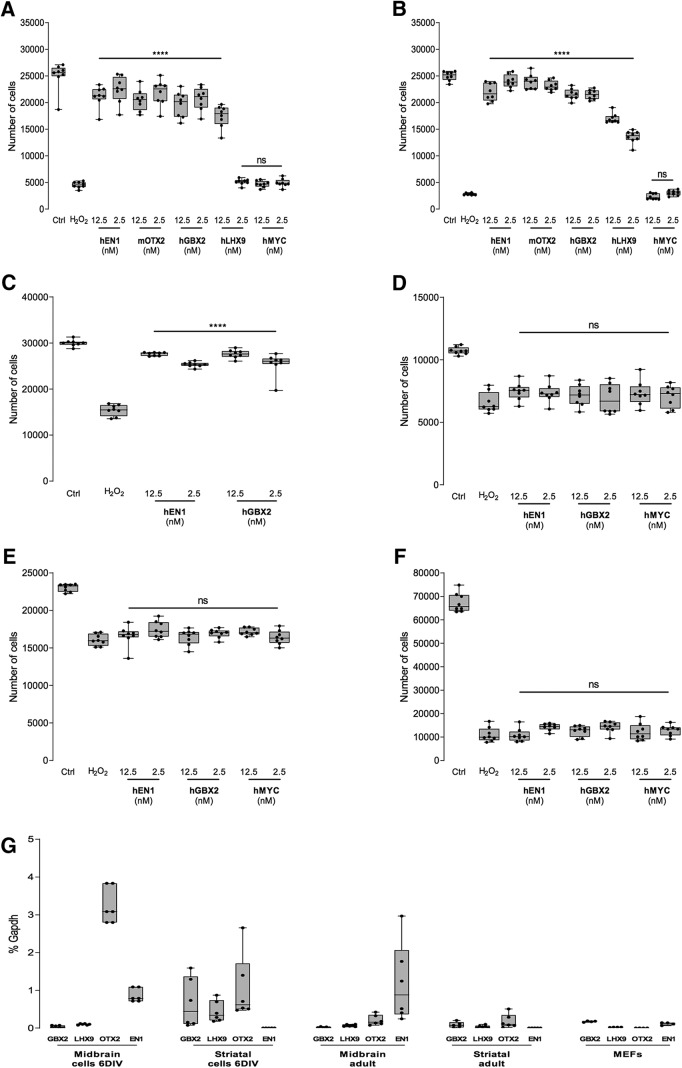
Homeoproteins protect embryonic neurons but not non-neuronal cells in the LDH assay. ***A***, hEN1, mOTX2, GBX2, and hLHX9 protect embryonic ventral midbrain cells against H_2_O_2_ oxidative stress, while hMYC does not. ***B***, hEN1, mOTX2, GBX2, and hLHX9 protect embryonic striatal neurons against H_2_O_2_ oxidative stress, while hMYC does not. ***C***, hE1 and hGBX2 protect primary astrocytes against H2O_2_ oxidative stress. ***D***, hEN1, hGBX2, and hMYC do not protect fibroblasts against H_2_O_2_ oxidative stress. ***E***, hEN1, hGBX2, and hMYC do not protect HeLa cells against H_2_O_2_ oxidative stress. ***F***, hEN1, hGBX2, and hMYC do not protect macrophages against H_2_O_2_ oxidative stress. ***G***, qRT-PCR reveals the expression of *GBX2*, *LHX9*, and *OTX2* in embryonic striatum, and *LHX9*, *OTX2*, and *EN1* in ventral midbrain. ns, nonsignificant, ****p < 0.0001.

Mouse EN1, hEN1, and mouse or chEN2 are neuroprotective toward midbrain dopaminergic cells *in vitro* and *in vivo* (Sonnier et al., 2007; Alvarez-Fischer et al., 2011; Rekaik et al., 2015). Protection was also observed for OTX2 on DA midbrain cells *in vivo* and on RGCs *in vitro* and *in vivo* (Sonnier et al., 2007; Alvarez-Fischer et al., 2011; Torero-Ibad et al., 2011; Rekaik et al., 2015). This raised the possibility that protective activity may be a property shared among a number of HPs. To determine whether protection against oxidative stress is shared by several HPs from different classes, mOTX2, hLHX9, hGBX2, and chEN2 were compared with hEN1 in a single experiment with embryonic ventral midbrain neurons. Figure 2*A* demonstrates that the four HPs provided significant protection against 50 mm H_2_O_2_ at 12.5 nm. Only hLHX9 at 2.5 nm failed to protect embryonic ventral midbrain neurons from H_2_O_2_ oxidative stress-induced cell death.

In contrast with the four HPs tested, a cell-permeable human MYC (hMYC) provided no protection (Fig. 2*A*). In addition, protection by all HPs, but not hMYC, was also observed with striatal embryonic neurons (Fig. 2*B*). This suggests that protection against oxidative stress may be specific to HPs with little neuronal subtype specificity. The fact that both striatal and midbrain neurons were protected, and the absence of HP specificity led us to use hEN1 and hGBX2 to verify whether they protected non-neuronal cells, including primary astrocytes, primary MEFs, HeLa cells, and primary mouse macrophages. One hundred mm H_2_O_2_ killed ∼50% of primary astrocytes, while 12.5 nm hEN1 or hGBX2 completely protected against this oxidative stress (Fig. 2*C*). Figure 2 also illustrates that although MEFs (Fig. 2*D*) and HeLa cells (Fig. 2*E*) are more resistant to H_2_O_2_-induced oxidative stress than neurons (Fig. 2*A*,*B*) or macrophages (Fig. 2*F*), none of the non-neural cells are protected by the two tested HPs or by cell-permeable c-MYC.

To verify whether this large HP spectrum was related to unspecific HP expression in culture conditions, we compared the expression of GBX2, LHX9, OTX2, and EN1 in 6 DIV cultures and adult tissues using qRT-PCR. Figure 2*G* illustrates that MEFs in culture express none of the HPs, and that, in the embryonic cultures, GBX2 is expressed in striatal neurons only, LHX9 and OTX2 in midbrain and striatal neurons and EN1 in midbrain neurons only. For comparison, OTX2 is expressed in adult midbrain and striatum, and EN1 is expressed in adult midbrain, whereas LHX9 and GBX2 are barely expressed in the two structures (Fig. 2*F*). These data demonstrate that a given HP can protect neurons that do not normally express it. For example, striatal neurons are protected from oxidative stress by EN1, yet they do not express it. Similarly, midbrain neurons are protected by GBX2, which they do not express.

### Homeoproteins protect midbrain embryonic neurons against DNA damage

**Figure 3.**
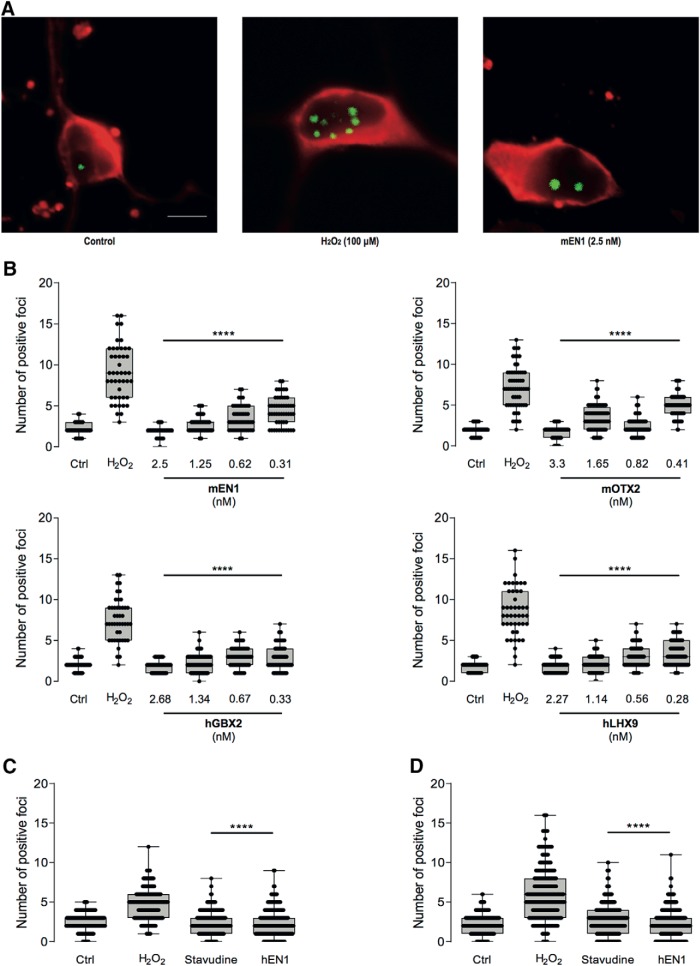
HPs reduce DNA breaks after H_2_O_2_. ***A***, Cultures of E14.5 ventral midbrain neurons (red) untreated (control) show few bright γH2AX foci (green), while those treated with 100 μm H_2_O_2_ have numerous foci and those pretreated with mEN1 have only a few. ***B***, Quantification of γH2AX foci. H_2_O_2_ increases the number of foci from ∼1-2 per neuron to ∼8. mEN1, mOTX2, hGBX2, and hLHX9 reduce the number of foci in a dose-dependent manner. ***C***, ***D***, Inhibition of reverse transcriptase activity protects against H_2_O_2_ oxidative stress in midbrain (***C***) and striatal neurons (***D***). In the control condition, few γH2AX foci are observed in embryonic midbrain neurons, while those challenged with 100 μm H_2_O_2_ show multiple DNA damaged foci. Pretreatment with 10 μm stavudine or 2.5 nm hEn1 completely blocks the formation of DNA damage foci. ****p < 0.0001.

Oxidative stress causes a number of changes in cell physiology, among which is the production of DNA breaks. In studies of neuroprotection of ventral midbrain neurons *in vivo*, Rekaik et al. (2015) observed that ENGRAILED reduces the number of anti-γH2AX-stained DNA damage foci induced in the nuclei of mDA cells exposed to 6-OHDA. To examine whether this is also the case in the present *in vitro* conditions and for the four HPs studied, embryonic midbrain neurons were cultured for 6 DIV, treated with the mEN1 at a 2.5 nm concentration for 24 h, and exposed for 1 h to 100 μm H_2_O_2_. The cells were fixed, and γH2AX foci were revealed by immunocytochemistry in neurons identified by βIII tubulin labeling. Without H_2_O_2_, neurons had only one or two γH2AX foci while H_2_O_2_ increased the number of foci about fourfold. Pretreatment with mEN1 reduced the number of γH2AX foci as illustrated in Figure 3*A*. As quantified in Figure 3*B*, the reduction in γH2AX foci was dose dependent for mEN1, hLHX9, hGBX2, and mOTX2 at concentrations ranging from 2.3/3.3 to 0.3/0.4 nm depending on the HP. Thus, each of the HPs tested protects neurons from oxidative stress, promotes their survival and reduces the level of DNA damage caused by H_2_O_2_.

Mobile element LINE-1 (L1) expression by midbrain neurons is increased by oxidative stress *in vitro* and *in vivo* and the endonuclease encoded by L1 ORFp2 (open reading frame 2) is in part responsible for the breaks (Blaudin de Thé et al., 2018). Accordingly, the protective activity of hEN1 is due to its ability to repress oxidative stress-induced LINE-1 overexpression (Blaudin de Thé et al., 2018). Here, EN1 and the reverse transcriptase inhibitor stavudine used as a LINE-1 antagonist protected the oxidative stress-induced formation of DNA brakes in midbrain neurons. This led us to compare the effects of an overnight pretreatment by 12.5 nm EN1 or 10 μm and stavudine on the number of γH2AX foci in midbrain (Fig. 3*C*) and striatal cell cultures (Fig. 3*D*) following a 1 h incubation with 100 μm H_2_O_2_. Figure 3, *C* and *D*, illustrates that 100 μm H_2_O_2_ increased the number of γ-H2AX foci by about twofold in embryonic striatal and midbrain neurons compared with control and that stavudine at 10 μm significantly reduced the foci to the same extent as hEN1 at 12.5 nm .

### hEN1 protects a dopaminergic cell line against 6-OHDA toxicity

**Figure 4.**
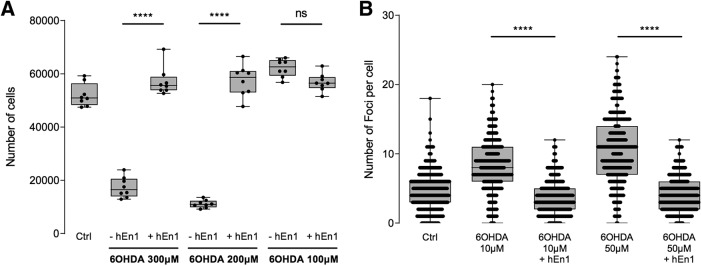
hEN1 protects immortalized human dopaminergic neuronal precursors, LUHMES cells, against H_2_O_2_ oxidative stress by LDH assay. ***A***, Three hundred and 200 μm 6-OHDA reduce the number of LUHMES cells surviving, while 50 nm hEN1 completely protects against this oxidative stress. ***B***, Quantification of γH2AX foci. 6-OHDA increases the number of foci by twofold to fourfold. Preincubation with hEN1 reduces the number of foci to the control level. ns, nonsignificant, ****p < 0.0001.

ENGRAILED and OTX2 protect mesencephalic dopaminergic neurons *in vivo* against an oxidative stress induced by 6-OHDA (Rekaik et al., 2015). To verify whether this is also the case *in vitro* and to follow the DNA protection activity, we used immortalized human dopaminergic neuronal precursors, LUHMES cells, that express the DA transporter and are thus sensitive to 6-OHDA (as opposed to midbrain neurons in which the mDA neurons constitute a minority of the cell population). Figure 4 demonstrates that LUHMES cells are sensitive to the toxin at 200 and 300 μm concentrations and entirely protected by a preincubation with 50 nm hEN1. DNA breaks were also followed at lower 6-OHDA concentrations, which induce breaks without provoking rapid cell death. As shown in Figure 4*B*, hEN1 reduces the number of breaks, confirming its protective activity in the 6-OHDA oxidative stress paradigm.

## Discussion

Exogenous ENGRAILED protects DA neurons *in vitro* against MPP^+^ and rotenone, and *in vivo* against 6-OHDA, MPTP, A30P α-synuclein, and progressive degeneration associated with the loss of one *En1* allele (Sonnier et al., 2007; Alvarez-Fischer et al., 2011; Rekaik et al., 2015; Thomasson et al., 2019). OTX2 promotes the survival of adult dissociated RGCs *in vitro*, protects RGCs *in vivo* against NMDA excitotoxicity and mDA neurons against 6-OHDA (Torero-Ibad et al., 2011; Rekaik et al., 2015). This similar prosurvival activity of two distinct transcription factors of different HP families led us to develop an *in vitro* assay to assess the ability of several HPs belonging to different classes to protect embryonic neurons against cell death and DNA damage caused by H_2_O_2_ oxidative stress. EN1, EN2, and GBX2 are members of the ANTENNAPEDIA class, OTX2 belongs to the PAIRED class, and LHX9 is part of the LIM class of HPs (Boncinelli, 1997).

The present results show that ENGRAILED internalization and high-affinity DNA binding properties are necessary for its neuroprotective activity. This is in accord with previous results showing that when the WF at positions 85 and 86 in OTX2 (thus, in positions 48 and 49 of its homeodomain) are mutated to YL, OTX2 loses its ability to be internalized and its neuroprotective activity for RGCs *in vitro* and *in vivo* (Torero-Ibad et al., 2011). The requirement for high-affinity DNA binding suggests that survival activity implies transcriptional regulation and not signal transduction through a cell surface receptor. This does not preclude activity at several other levels, including the regulation of protein synthesis or the maintenance of a healthy heterochromatin, as demonstrated in studies on the protection of SNpc mDA neurons by ENGRAILED (Alvarez-Fischer et al., 2011; Stettler et al., 2012; Rekaik et al., 2015; Thomasson et al., 2019). Whether these conclusions apply to all other HPs tested here is an open question.

DNA break-induced signaling such as the phosphorylation of the histone variant H2AX (γH2AX) is required for transcriptional elongation in healthy cells. In this case, γH2AX accumulates at gene transcription start sites (TSSs) during Pol II pause release (Bunch et al., 2015). However, there are clear differences between the latter situation and γH2AX-marked double-strand breaks (DSBs) induced by damaging conditions, including oxidative stress. In TSSs, γH2AX accumulation is condensed within the transcribed units only, and there is no spread outside the boundaries of the transcribed genes. In contrast, γH2AX accumulation due to DNA damage can spread over megabases in both directions from DSB sites. Here, the oxidative agents H_2_O_2_ and 6-OHDA significantly increased the number of γH2AX foci in embryonic neurons or LUHMES cells, respectively. Pretreatment with EN1, OTX2, GBX2, or LHX9 (embryonic neurons) or EN1 (LUHMES cells) prevented the formation of DSBs. Interestingly, another homeobox gene, HOXB7, enhances nonhomologous end-joining DNA repair *in vitro* and *in vivo* (Rubin et al., 2007), providing additional support for the involvement of homeoproteins in DNA break repair.

Homeoproteins of different species (chicken, mouse, and human) protect mouse embryonic neurons against oxidative stress, suggesting an evolutionary conservation of their protective activity that parallels their structure conservation (Banerjee-Basu and Baxevanis, 2001; Holland and Takahashi, 2005; Holland, 2013). The HPs tested here were all effective on neurons originating from the mesencephalon and telencephalon, two structures of different ontogenetic origins, thus expressing different repertoires of developmental genes. ENGRAILED and OTX2 expressed in the midbrain provide protection to striatal neurons and, conversely, GBX2 and LHX9 that are expressed in striatum are effective in providing protection to ventral midbrain neurons. These results raise the possibility that neuroprotective activity may be common to HPs in a non-region-specific manner. Interestingly, cell-permeant MYC, a transcription factor of the basic helix-loop-helix family with major roles in cell cycle progression, apoptosis, and cellular transformation, showed no neuroprotective effect against oxidative stress induced by H_2_O_2_.

In contrast with their neuroprotective activity for terminally differentiated nonproliferating embryonic neurons, astrocytes, and the LUHMES immortalized human dopaminergic neuronal precursor cell line, none of the HPs tested was able to protect HeLa cells, primary macrophages, or primary fibroblasts from H_2_O_2_ oxidative stress. Because all tests on non-neural cells were performed in the presence of the Ara C mitotic inhibitor and because the LUHMES cells are protected by hEN1, it is unlikely that the absence of protection in non-neural cells is due only to their proliferative status. A possible explanation, based on the importance of hEN1 in mDA neurons in chromatin remodeling (Rekaik et al., 2015) is that the chromatin structure of the proliferative cells tested here is sensitive to HP expression. Alternatively, but not mutually exclusive, cofactors required of HP protection might not be available in these non-neural cells. Finally, oxidative stress increases LINE-1 expression and retrotransposition events, increasing DNA damage. ENGRAILED reduces dopaminergic neurodegeneration by repressing LINE-1 expression *in vivo* (Blaudin de Thé et al., 2018). The results here extend the protective effects of the reverse transcriptase inhibitor stavudine to embryonic midbrain cells and striatal cells *in vitro*.

All in all, our results show that EN1, EN2, OTX2, GBX2, and LHX9 representing three different classes of HP transcription factors can protect embryonic cultured neurons from two ontogenetically diverse brain regions against H_2_O_2_-induced oxidative stress. The similar neuroprotection by ENGRAILED proteins from different species (i.e., chicken, mouse, and human) demonstrates a strong evolutionary conservation of this activity. The ENGRAILED genes of vertebrates and insects arose as independent duplication of an ancestral *ENGRAILED* gene (Dolecki and Humphreys, 1988), and this duplication occurred after the divergence of echinoderms and vertebrates but before the divergence leading to birds and mammals some 310 million years ago (Logan and Joyner, 1989; Benton, 1993; Kumar and Hedges, 1998). This suggests that ENGRAILED neuroprotective activity arose before the separation between birds and mammals. More strikingly, HPs can compensate between classes as shown by the fact that OTX2 of the PRD class is able to compensate for EN1-dependent neuronal loss *in vivo*, although ENGRAILED belongs to the ANTP-class (Di Giovannantonio et al., 2013). Thus, neuroprotective activity arose before the divergence of ANTENNAPEDIA and PAIRED classes of HPs. The early emergence of HP protection and the selective pressure to maintain it over hundreds of millions of years underscores the importance of this HP activity.

## Synthesis

Reviewing Editor: Julie Andersen, Buck Institute Decisions are customarily a result of the Reviewing Editor and the peer reviewers coming together and discussing their recommendations until a consensus is reached. When revisions are invited, a fact-based synthesis statement explaining their decision and outlining what is needed to prepare a revision will be listed below.

Long known, Homeoproteins are large class of proteins to be involved in variety of physiological functions. However the property of these class of proteins to reverse the physiology, particularly in the nervous system during pathology associated conditions for cellular function and survival had become one among the attractive targets for therapy in recent years. Acute conditions like early stages of Parkinson's disease had shown reduced levels of these proteins. The promising effect have been also demonstrated in-vivo. However the basic understanding is yet to be well studied. This manuscript explains the role of homeoproteins under oxidative stress and free radical induced DNA repair to promote neuronal survival in a class of neurons.

The authors should revise “Table 1” carefully check if they have added all informations, labelling. For e.g. (Line 193; Table 1) in Table Figure 1-A H2O2 vs 25pM should be H2O2 vs 2.5pM to match with Fig.1A (or vice-versa, not sure which is correct). Also for Fig. 1-D hEN1 only group is not mentioned in the table.

1. Authors are advised to revise with “Journal language”. At some places the flow of the text seems to be a normal spoken English.

2. Through out the manuscript there are several concerns to be explained. I will list out the following in order.

a. In the manuscript (see Line 5) these proteins are involved in protecting adult cells. However the authors used embryonic cells with regular neuronal differentiation factors. It might make the explanations strong if the authors could explain or how they could consider the embryonic neuronal model to the adult neuron.

b.I believe the authors particularly selected region specific tissues to culture in-vitro for the whole experiments. However they should have emphasised neuronal specific marker (Thyrosine Hydroxilase) instead of Tuj1 (line 178 for reference). Which might make issues in data interpretation during foci count. An immunostaining image would be better, since dopamine or ventral neuronal specific cues are not available for almost 6days in-vitro.

c.However not a big issue, astrocytes could have been used instead of mouse fibroblasts to support more strongly the homeoproteins in the “brain fibroblasts” or it might have also made us to understand what happens to other non-neuronal cells during the similar condition in brain oxidative stress.

d. Line 70, “cell death” should be re-considered.

e. Authors consider H2O2 and 6OHDA as stressors. Throughout the text a consistency of mentioning the name of the compound next to oxidative stress might be making the readers more clear to the context.

f. Authors had chosen two different doses for H2O2 (50uM and 100uM) for different homeoproteins. It is not consistent. (Line 153, 290, 308).

e. It raises concern why authors were not interested to examine mature neurons (LUHMES can be differentiated by GDNF, according to ATCC). Ofcourse the authors used embryonic neurons that match with the current model. However, the story or importance is more towards mature neurons.

f. Line 199. Since authors show only some protein for some experiments all five protein names list would make the readers to understand.

g. Line 204, pretreatment time? At some places the treatment times were changed (line 153 and 224).

h. rephrase line 224, 280.

i. Figure not match with main text Line 309 - 310.

j. Reference year order for DiNardo AA et. al.,

k. Figure legend mention the assay name (LDH assay)

l. Figure 3 A, there is some insert alphabets in the figure.

Over all the information available add on to the community with informations during oxidative stress induced DNA damage and repair associated neuroprotection. This is also applicable out of the field for researchers interested to study radiation oncology to understand the damage induced neuron death caused by DNA break.
