# Erratum: Vargas Abonce et al., Homeoprotein Neuroprotection of Embryonic Neuronal Cells

**DOI:** 10.1523/ENEURO.0227-20.2020

**Published:** 2020-06-23

In the article “Homeoprotein Neuroprotection of Embryonic Neuronal Cells,” by Stephanie E. Vargas Abonce, Mélanie Leboeuf, Alain Prochiantz, and Kenneth L. Moya, which published online on August 26, 2019, the author contributions were incomplete. It should be listed that Stephanie E. Vargas Abonce and Mélanie Leboeuf contributed equally to this work. This correction does not affect the conclusions of the paper. The equal contributions of S.E.V.A. and M.L. have been corrected online.

